# Notices

**Published:** 1863-08

**Authors:** 


### NOTICES.

Arthur's Home Magazine, $2 a year, 323 Walnut street, Philadelphia, Pa., is the most unexceptionable periodical in this country for general family reading; *always* on the side of purity, virtue, and a blameless life. *No* household can take it without being instructed and made more happy by its chaste and pure teachings.

Blackwood's Magazine, $2 a year, reprinted by Leonard Scott & Co., Walker street, New-York. If taken with the four reviews, *Edinburgh*, *Westminster*, *North British*, and the *London Quarterly*, all are furnished for $10 a year. These publications are written for by the best and most finished scholars in Great Britain, and merit a wide circulation, especially among gentlemen of wealth and education.

Godey's Lady's Book, $3 a year, Philadelphia, Pa., has not only outlived, but has surpassed all the pictorial monthlies of its kind. Nothing short of a very wide circulation could sustain an issue got up with such a great expense, with such an elaboration of embellishments, fashion-plates, and colored engravings; to say nothing of the practical utility of its reading matter in reference to domestic matters, on cookery, crochet-work, chemistry, fashions, rural items, plans, illustrations, etc., with its health department by its permanent contributor, Dr. J. S. Wilson.

Harper's Weekly, illustrated, $2 a year. *Harper's Monthly*, which alone is $3. Both are furnished for $4 a year.

Portrait Monthly of the New-York *Illustrated News* of the "Men of the Times." Single numbers, 10 cents; or $1 a year. Number one contains the likeness of twenty-five men of eminence, North and South. We trust it will have a wide sale. There is not a likeness in the last issue which is not instantly recognized.

We willingly call attention to the *American Phrenological Journal*, issued monthly, at $1.50 a year. We always open it with interest, as an important key to the understanding of human character, worth more than any other item of knowledge toward securing in life pecuniary success or a great name. Published by Fowler & Wells, 308 Broadway, New-York.

Braithwaite's Retrospect is published semi-annually for $2 a year, an 8vo of 650 pages, semi-annual numbers, $1.25 each, by W. A. Townsend, No. 30 Walker street, New-York. Each issue contains all that is new in medicine and surgery for the previous six months, an epitome of medical progress throughout the world, and is of great practical value to every physician.

The American Tract Society, 28 Cornhill, Boston, and No. 9 Bible House, New-York, have issued in comely style the following books for children's reading: *Lessons from Insect Life*, which adults can not peruse without being edified in heart as well as in mind; *The Happy Home;* or, The Story of Annie Lyon. A touching narration. *Harry*, containing "The Two Homes," "The Village Store and Sign," "The Minister," etc. *The Babes in the Basket;* or, Daph and her Charge. A true narration connected with the massacres of St. Domingo. It will intensely interest every reader; an impressive lesson of single-heartedness and fidelity, and its reward on a dying-bed. *The Pilgrim Path*, being interesting incidents in the experience of Christians, with earnest words from many who love the Lord. This is just such a book as ought to be multiplied by the million, for humble growing Christians can feast and feed upon it every day. *Following after Jesus*; or, a Memorial of Susan Maria Underwood, by Mrs. Eliza H. Anderson. Christian biography, conscientiously and truthfully written as this volume is, is one of the best educators to a Christian life next to the Bible, and books like these can be profitably read and practically used by all—rich and poor, young and old.

Moses and the Pentateuch: A Reply to the Bishop of Colenso, by the Rev. W. A. Scott, D.D., late Moderator of the O. S. Presbyterian Church in the United States. Just published by William Freeman, 102 Fleet street, London, and Hugh Barclay, 26 Temple street, Birmingham. This new contribution to Christian literature by the reverend author, formerly of New-Orleans and late the eminent and successful pastor of the Calvary Church of San Francisco, is an additional evidence of the industry and ability of an eminent clergyman. The London *Christian World* says of it, that it "is evidently composed by a man of superior intellectual attainments and historical knowledge;" and the Glasgow *Herald* (Scotland) testifies: "It is bold and decided in its tone, and will speak for itself as no ordinary orthodox exposition, and a valuable addition to theological literature." The many friends of Dr. Scott, and especially the large number of families and gentlemen in

New-York City who once sat delighted under his ministry in New-Orleans, (the editor being one,) will be greatly pleased to learn that their old pastor and friend, with his family, except a gallant son, an officer in the Union army, have reached New-York in health and safety. Of course, a man of such ability would be caught up in a week after his return to our shores, and he is supplying the pulpit of the Rev. Dr. Van Wyck, in Brooklyn, until September. Thrice fortunate will be that people who will haste to secure his permanent ministrations.

Post-Office Notice, Official.—The fifth sub-division of the forty-second instruction under the new Post-Office law is hereby amended by striking out the word *twelve* and inserting *thirty-two* before ounces, so that it shall read as follows: "The weight of packages, of seeds, cuttings, roots, and scions to be franked, is limited to thirty-two ounces." By order of the Postmaster-General,

Alex. W. Randall,

Washington, July 6th, 1863. First Asst. Postmaster-General.

In order that our readers, who are not officially connected with the mail service, may have a full understanding of the changes in postal matters effected by the new law, which went into operation on the first of the present month, we give below a condensed summary of those of its provisions of which it is necessary for persons using the mails to "take due notice and govern themselves accordingly:"

1. The rate of postage on all domestic mail letters to be carried any distance within the United States is now three cents per half ounce or fraction thereof, to be prepaid by stamps. The former rate of ten cents to California, Oregon, and Washington Territories is abolished.

2. All local or drop-letters must hereafter be prepaid by stamps, at the rate of two cents for every half ounce or fraction thereof, instead of one cent each, as heretofore.

3. The postage on transient newspapers and periodicals, sent in one package to one address, is now two cents for each four ounces or each fraction thereof, to be prepaid by stamps; on books, double that rate. The postage on single transient newspapers not weighing over four ounces is now two cents.

4. The rate of postage on circulars is now as follows: Three or any less number may be sent, unsealed, to one address, at the single rate of two cents, and in that proportion for a greater number, adding one rate for every three circulars directed to one address. They can no longer be sent at the former rate of one cent each. No extra charge is now made for business cards stamped or printed on the envelopes of circulars.

5. The former carriers' fee of one cent on each letter delivered is abolished. Hereafter, carriers collect nothing, except such unpaid postage as may be due on the letters delivered by them.

6. The extra one-cent stamp formerly required on all letters deposited in lamp-post boxes and branch stations is no longer necessary.

7. All communications to any officer or department of the Government, (including the President,) written by a private citizen, whether on "official business" or otherwise, must now be prepaid by stamps.

8. A fee of twenty cents (instead of five, as heretofore) must hereafter be paid on each registered letter in addition to the postage.

9. The letter can not be forwarded without a charge of extra postage when it has once been mailed according to its original address.

There is now no use for one-cent postage stamps.

